# India's oral health outlook: challenges, economic impact and need for preventative strategies

**DOI:** 10.3389/fdmed.2025.1544899

**Published:** 2025-04-09

**Authors:** Prashant Narang, Ashok Dhoble, Manu Mathur, Salaj Rana, Steve Mason, Asif Ali

**Affiliations:** ^1^ Medical & Scientific Affairs, Haleon (Erstwhile GlaxoSmithKline Consumer Healthcare), Gurgaon, Haryana, India; ^2^ Indian Dental Association (Head Office), Mumbai, India; ^3^ Dental Public Health and Primary Care, Institute of Dentistry, Barts and the London School of Medicine and Dentistry, Queen Mary University of London and Head: Health Policy—Public Health Foundation of India, New Delhi, India; ^4^ Division of Non-Communicable Diseases, Indian Council of Medical Research (ICMR), New Delhi, India; ^5^ Oral Health Research and Development, Haleon (Erstwhile GlaxoSmithKline Consumer Healthcare), London, United Kingdom

**Keywords:** oral health, quality of life, economic burden, health policy, India, dental care, preventive care

## Abstract

**Objective:**

This review explores the economic and behavioral dimensions of oral health in India, emphasizing the economic burden, treatment-seeking behaviors, and policy-level interventions needed to improve oral health outcomes.

**Methods:**

A mixed-method approach was employed, integrating primary data from the Oral Health Observatory (OHO) project (2016–2020) and a systematic review of secondary literature. The OHO project surveyed dental clinic patients using mobile app questionnaires, focusing on oral health behaviors, barriers, and outcomes. Secondary data were analyzed from PubMed, MEDLINE, and Embase databases to assess the economic implications of oral health care in India.

**Results:**

The OHO data revealed a steady decline in routine dental visits after the age of 35–44 years, while emergency visits persisted across age groups. Gender disparities were observed, with men reporting higher dissatisfaction, greater oral pain, and work disruptions compared to women. Only 44.7% of participants brushed twice daily, and 36.7% brushed before bed. Secondary findings reveal that untreated oral diseases have a higher economic impact than preventive measures. This highlights the need for consumer awareness, strategic policies, and sustainable healthcare frameworks.

**Conclusion:**

India faces significant challenges in oral health due to low awareness, insufficient infrastructure, and economic barriers. Strengthening preventive strategies, increasing public-private partnerships, and integrating oral health education into primary care can dramatically reduce costs and improve population outcomes. A shift from reactive to preventive care is essential for ensuring equitable and sustainable oral health solutions.

## Introduction

Oral health is a fundamental component of holistic well-being, encompassing physical, mental, social, emotional, and spiritual health (1). As a critical aspect of holistic wellness, oral health means not only the absence of dental diseases, but it also plays a significant role in shaping overall well-being and quality of life. Poor oral health can lead to chronic pain, low self-esteem, and social stigma, which negatively affect mental and emotional health. Despite its profound influence, oral health is often overlooked within healthcare systems worldwide (2, 3). Many oral health conditions, though chronic, are preventable through basic care interventions such as regular brushing, flossing, and routine dental check-ups which help prevent issues like cavities, gum disease, and infections. Therefore, addressing disparities in oral healthcare access and integrating it into primary healthcare systems are crucial for promoting well-being (4, 5). Recognizing this, global health authorities, including the U.S. Department of Health and Human Services and World Health Organization (WHO) have set ambitious targets to improve oral health by 2030, with numerous initiatives already underway to raise awareness, increase access to care, and integrate oral health into broader health agendas (6, 7). In India, the prevalence of oral health conditions is alarmingly high, driven by multifactorial challenges. While 68.84% of the population lives in rural areas, the majority of dental professionals prefer to work in urban regions, leading to acute workforce shortages even in cities provide service in the urban areas (8). Despite efforts by organizations like the Indian Dental Association (IDA) to meet public needs, the current infrastructure and manpower within primary healthcare services fall short of delivering comprehensive oral care (9, 10).

The dental workforce in India predominantly focuses on treatment and emergency services, with limited attention to preventive dentistry. This trend stems from factors such as insufficient public awareness of preventive care, a lack of integration of oral health into the broader concept of holistic health, high treatment costs, and inadequate policy interventions (11). Consequently, untreated oral health conditions often worsen over time, necessitating professional intervention at advanced stages. This delay not only increases treatment costs but also imposes a significant economic burden on individuals and the workforce, as people lose productive workdays due to oral health issues (11).

Policy interventions are critical to creating a sustainable oral health framework in India. Efforts must focus on raising awareness, improving access to care, and empowering communities. Current initiatives aim to enhance public awareness about the impact of poor oral health on quality of life, improve basic infrastructure, expand insurance coverage for oral health services, and address the uneven distribution of dental professionals. Additionally, increasing the utilization of oral health services is a key priority within the country's oral health reform (12). Utilization studies play a critical role in shaping oral health policies and understanding treatment-seeking behavior. By addressing the frequency care needs and associated costs relative to population needs and workforce capacity, policymakers can identify cost-effective strategies to improve service delivery (13). However, Indian studies addressing treatment-seeking behavior and the economic burden of oral diseases remain scarce.

This review seeks to address this gap by integrating primary and secondary data to provide a comprehensive understanding of India's current oral health landscape. The primary objective is to highlight the economic impact of deteriorating oral health and offer insights that inform strategies for prevention and management. Additionally, this study emphasizes the urgent need for policy interventions to improve access to care, promote preventive services, and establish a sustainable oral health service framework.

## Methods

This study adopted a mixed-method approach, wherein data were gathered from both primary and secondary sources (13).

### Primary data analysis

The Oral Health Observatory (OHO) project was carried out in India between 2016 and 2020 to examine oral health-related habits, attitudes, behaviors, barriers, and outcomes within the Indian context. This project was carried out jointly by the World Dental Federation (FDI) and the Indian Dental Association (IDA), with analytical support from the University of Sheffield, United Kingdom, and sponsored by Haleon (erstwhile GlaxoSmithKline Consumer Healthcare). The study employed a cross-sectional, analytical observational design, utilizing a mobile application with two questionnaires‒one for patients and another for dentists‒to gather data on clinical oral health (14).

### Patient sampling and recruitment

A modified systematic sampling method was used to select participants from dental clinic patients. Each working day, one patient was surveyed based on their arrival order. If a patient declined to participate, the next one was invited. This simple technique minimized errors and dentist dropout rates. Each dentist surveyed 50 patients, all of whom provided informed consent and were residents of the study country. For participants under the age of 12 years, proxy consent was obtained from parents. Consent was digitally recorded via the mobile application, and patients were provided with an information sheet. A preliminary descriptive analysis was performed to detail survey variable and segment the data by country and key demographics such as age and gender.

### Secondary search analysis

To gain insight into the economic implications of the survey findings, we conducted a systematic literature search to identify relevant articles which was carried out using PubMed, MEDLINE, and Embase databases. The following key search terms were used “Oral health OR Dental health OR Oral hygiene OR Dental care OR Oral healthcare OR Oral well-being OR Dental well-being OR Oral condition OR Dental status” AND “Economic burden OR Economic health OR Financial health OR Fiscal health” AND “India”. Non-English literature was excluded. Additionally, data from medical and economic health websites seamlessly integrated with our current findings to provide a comprehensive overview of the economic aspects associated with the oral health issues discussed in the present study.

## Results

### Primary research results

**Figure 1:**
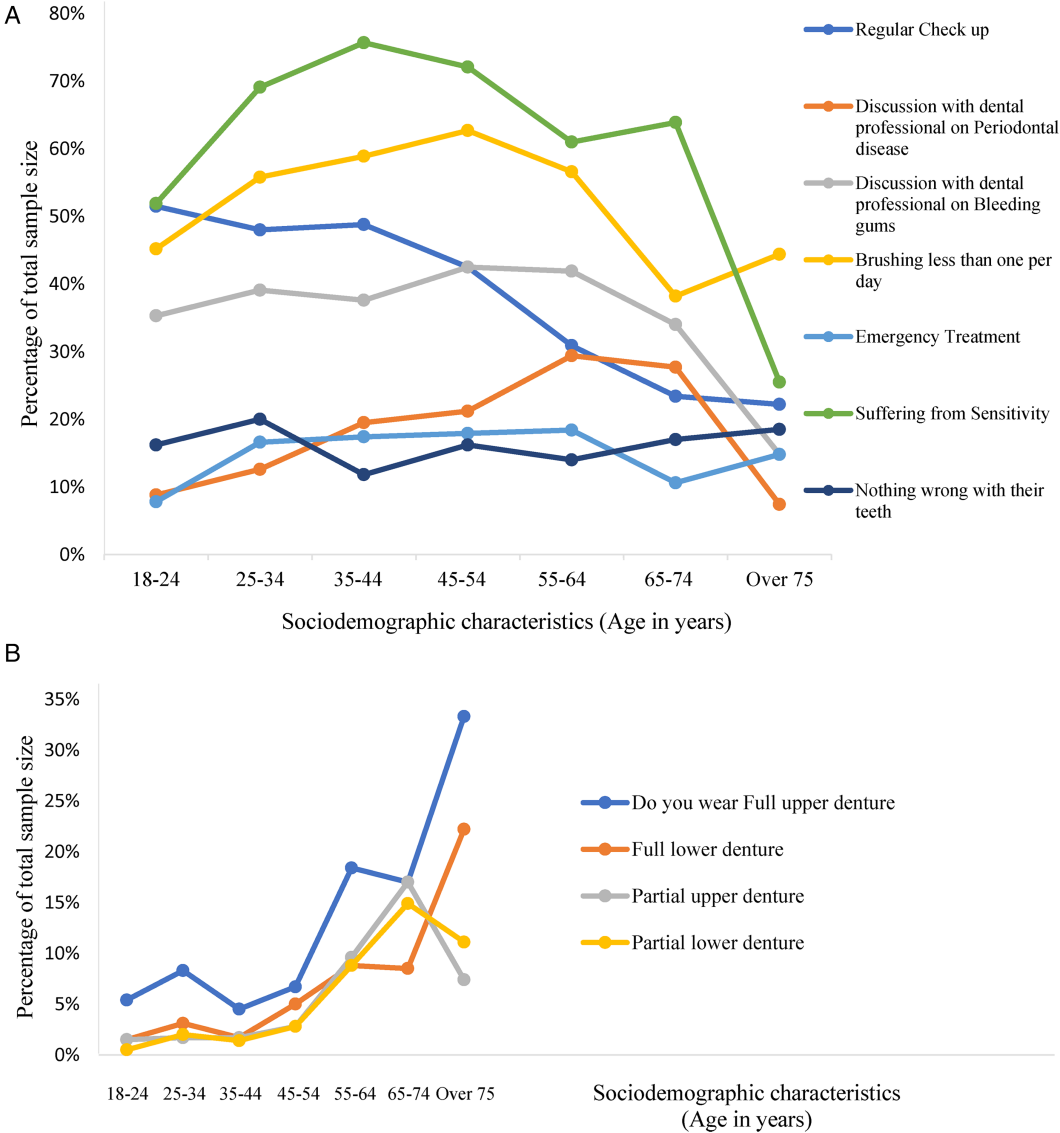
**(A)** Age driven oral health complications and oral health care seeking behavior pattern. [Participants had a choice to select more than one variable (option), each response category is treated as a variable, calculated as percentage of total sample size as hundred percent (100%)]. **(B)** The relationship between age and use of denture. [Participants had a choice to select more than one variable (option), each response category is treated as a variable, calculated as percentage of total sample size as hundred percent (100%)].

Data from the OHO project revealed that the prevalence of an oral health checkup was highest (51.5%) among younger participants in the 18–24 years age group and decreased steadily with age, reaching its lowest (22.2%) in older participants over 75 years. As age increased, the frequency of in-clinic conversations about bleeding gums and periodontal health increase until the 55–64 years age group (Figure 1A). An important insight from the data is the notable decline in routine oral health visits after the age of 35–44 years, while emergency visits remained steady between the age groups of 25–64 years.

The age group 55–64 years appears to be pivotal period for oral health, post which the incidence of partial dentures dips dramatically, with a corresponding and equally dramatic increase in the use of full dentures (Figure 1B). This could potentially relate to the transition from partial edentulism to complete edentulism within this age range. Emergency treatment was less prevalent in the 18–24 years age group (7.8%) compared to the 55–64 years age group (18.4%), as shown in Figure 1A. Similarly, the prevalence of sensitive teeth was lower in younger participants and increased with age. Interestingly, a significant proportion in the 24–35 years (20%), 55–64 years (14%), and 65–74 years (17%) age groups reported that they believed nothing was wrong with their teeth despite oral health issues (Figure 1A).

**Figure 2:**
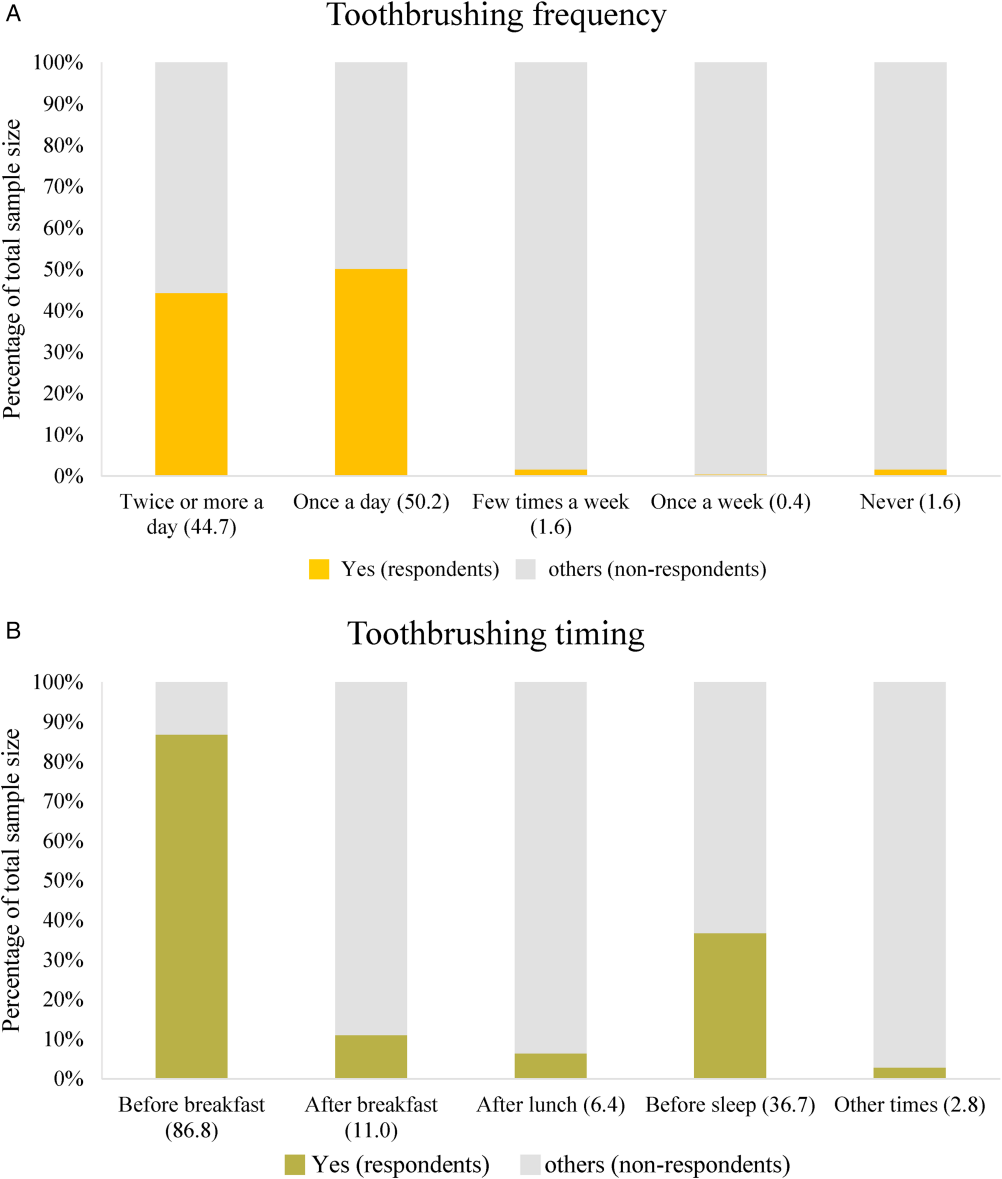
**(A)** Frequency of toothbrushing (oral hygiene habits). [Participants had a choice to select more than one variable (option), each response category is treated as a variable, calculated as percentage of total sample size as hundred percent (100%)]. **(B)** Timing of toothbrushing (Oral hygiene habits). [Participants had a choice to select more than one variable (option), each response category is treated as a variable, calculated as percentage of total sample size as hundred percent (100%)]. B, Billion; INR, Indian Rupee; M, Million; MoHFW, ministry of health and family welfare; NHM, national health mission.

Gender differences were also observed. The frequency of dental visits was slightly higher in women than men (92.0% vs. 90.3%), while men reported higher level of general dissatisfaction with life (28.8% vs. 25.9%). Men also experienced a higher prevalence of difficulty performing major work activities due to pain in the mouth, teeth, or dentures in the past 12 months compared to women (14.2% vs. 8.5%). Additionally, more men took time off work due to oral pain (18.2% vs. 11.4%) and reported difficulty speaking due to pain (19.1% vs. 9.3%). In terms of oral hygiene habits, the OHO data revealed that only 44.7% of participants brushed their teeth twice daily, and just 36.7% brushed their teeth before going to bed (Figures 2A,B).

### Secondary research results

The search yielded 24 articles, of which nine were excluded based on their abstracts. Full texts were obtained for the remaining 15 articles and upon further review, five articles were deemed ineligible due to irrelevant information. Finally, ten articles were selected for analyzing the economic impact of oral health care. Among these, six studies have shown the expenses related to both preventive and emergency dental visits, as summarized in Table 1 (15–20). Four studies have depicted the comparison between expenditure on oral healthcare with the projected costs of preventive dental care (21–24).

**Table 1:** Studies on oral health expenditure in India.

S. No	Author, Year, Reference	Study design and population	Objective	Expenditure on oral health care [All the cost in Indian national rupees (INR)]
1	Meenal et al. (15)	Questionnaire based observational study, 250 adults presenting to government dental hospitals and 250 adults presenting to private dental clinics across Chennai for their dental care.	To evaluate the out-of-pocket costs in the private and the public sector in the field of dentistry across Chennai, India	Expenditure on dental care every year • Visiting fee in private dental care: INR 140 • Procedure fees: INR 5,488 • Traveling cost for dental visit: INR 207.63 • On average, private sector patients spent for dental care every year INR 5,488.2 Preventive dental care (visiting fee) is cheaper than emergency care (procedure fee)
2	Desai et al. (16)	Logical explanation on calculation of direct and indirect cost for dental services provided at dental centre	To calculate the direct treatment and indirect costs for dental services provided at the dental centre.	Cost of different dental treatments: • Rotary RCT: INR 7,600 • Re-RCT: INR 10,900 • Extraction: INR 1,700 • Scaling: INR 3,300 • Porcelain-fused-to-metal (PFM): INR 7,900
3	Verma et al. (17)	Questionnaire based cross-sectional study on 860 participants in Durg, Chhattisgarh	To estimate the household expenditure on oral health care among people residing in Durg, Chhattisgarh	Expenditure on oral hygiene and dental treatment per year • Spend on oral hygiene for you and your family per year: INR 1,255.65 • Spend on the treatment of dental problem for you and your family since the last 1 year: INR 2,254 Average annual expenditure on you and your family: INR 4,368
4	Nobelika et al. (18)	Cross-sectional study based on interview schedule, 246 sanitary workers in Coimbatore, India	To find the patterns of oral health-care expenditure, factors associated with catastrophic dental health expenditure (CDHE), and knowledge about health insurance of sanitary workers in Coimbatore, India	Number of working days lost and catastrophic dental health expenditure • Number of working days lost due to dental illness in last 12 months: – One: 31 (12.6%) – Two: 37 (15%) – Three and above: 8 (3.3%) • 5.3% of the participants have spent more than INR 5,000/month • 11% have spent more than INR 2,000/month. • The reported prevalence of CDHE was 15.4%. During regular dental visits, dentist assess the dental health and identify areas of concern before they worsen.
5	Sunil et al. (19)	Based on consolidated Health Economic Evaluation Reporting Standards statement in dentistry	To calculate direct treatment and indirect costs for dental services provided at tertiary care referral dental centre.	Cost of different treatments in dental care • Rotary RCT: INR 7,624 • Re-RCT: INR 10,336 • Extraction: INR 1,706 • Scaling: INR 3,312 • Porcelain-fused-to-metal (PFM): INR 7,324 • Fillings: INR 1,756
6	Syamkumar et al. (20)	Questionnaire based observational study, 500 people residing in Kerala, India	To estimate the household expenditure on oral health care among people residing in Kerala, India	Expenditure on dental hygiene and dental treatment per year • Spend on oral hygiene for you and your family per year: INR 1,620 • Spend on the treatment of dental problem for you and your family since the last 1 year: INR 2,500 Average annual expenditure for you and your family: INR 4,120

**Expenditure on oral healthcare in 2019:** WHO highlighted the significant economic impact of oral diseases in India. The costs associated with treatment and prevention of these diseases posed a substantial financial burden on the country. This economic impact included both direct treatment costs and indirect costs, such as loss of productivity due to workdays missed. The total economic burden was reported to be 613.2 billion INR (USD 7.3 billion), reflecting the urgent need for more effective prevention and treatment strategies to mitigate these costs (21).

**Expenditure on projected preventive dental care in 2019:** The data indicated that an average household spent 1,255.65 INR (USD 15.6) annually on oral hygiene across 300 million households (17, 22). Preventive consultations provided by the government institute like AIIMS via its present healthcare framework are available at a cost of 10 INR (USD 0.125) per head annually, which indicates the potential cost-effectiveness of a well-established preventive care system (21). However, untreated oral diseases have a higher economic impact than preventive measures. This highlights the need for consumer awareness, strategic policies, and sustainable healthcare frameworks (23).

## Discussion

This study sheds light on the complex framework of oral health behaviors and the economic burden of dental care in India by utilizing OHO data from 2016 to 2020 and conducting a systematic literature review. The findings from OHO data suggest a decline in routine oral health visits after the age of 35–44 years, despite the persistence of emergency visits across age groups. This pattern indicates that many individuals may neglect preventive care as they age, relying more on urgent treatments. The age group 55–64 years emerges as a pivotal period for oral health, marked by the transition from partial to complete edentulism, which may explain the sharp increase in full denture use. During this period, periodontal health concerns and emergency dental treatments also rise, indicating a potential gap in earlier preventative care efforts. These findings align with a systematic review by the US Preventive Services Task Force, which observed that younger individuals tend to have more frequent oral health checkups compared to older adults (24). Physical limitation and the loss of dental coverage upon retirement contribute to older adults higher likelihood of requiring emergency care for oral health issues (24). The analysis also revealed notable gender differences, with men reporting higher levels of dissatisfaction with life, more severe oral pain, and higher rates of work-related disruptions due to dental issues compared to women. These disparities underscore the broader impact of oral health on quality of life and productivity. Additionally, only a minority of participants practiced optimal oral hygiene habits, which could contribute to the high prevalence of oral health issues reported across age groups. Health promotion strategies tailored to men, emphasizing the long-term benefits of preventive care, could help mitigate these disparities (25). Another key finding is the disconnect between self-perceived oral health and actual oral health conditions, particularly in the 24–35 and 55–64 years age groups. Many individuals believed their oral health was satisfactory despite having significant issues, indicating a lack of awareness or understanding of oral health risks. This underscores the importance of targeted public health education and outreach efforts to raise awareness of preventive care and early treatment (23). Economically, the burden of oral health issues is staggering. The estimated annual cost of INR 613.2 billion (USD 7.3 billion) includes both treatment expenses and productivity losses (26). In contrast, preventive oral care is remarkably cost-effective, requiring only 10 INR (USD 0.125) per person annually. These findings highlight the economic rationale for scaling up prevention-focused strategies (21). Investments in preventive care could alleviate the financial strain on individuals and the healthcare system, while significantly improving oral health outcomes. India has initiated several programs to improve oral health nationwide. For example, the Danta Bhagya Yojana has established 85 Muskaan clinics that provide free dentures to individuals aged 65 years and above. Other initiatives, such as the National Cancer and Tobacco Control Program and National Rural Health Mission, aim to bridge the rural-urban gap in oral healthcare (21, 27). However, the success of these initiatives remains limited by financial constraints, infrastructure challenges, insufficient public awareness, and inadequate budget allocation (28).

### Paediatric oral health

Paediatric oral health is a fundamental aspect of overall well-being, influencing child's nutrition, speech development, and self-esteem. One of the most common oral health concerns in children is early childhood caries (ECC), a severe form of tooth decay primarily caused by frequent exposure to sugary foods and drinks. Malocclusion, or misaligned teeth, is another prevalent issue that can impact speech and require orthodontic intervention. Gum disease and fluorosis, resulting from poor oral hygiene and excessive fluoride intake, respectively, are additional concerns that affect long-term dental health (29).

Preventive measures are crucial for maintaining children's oral health. Fluoride-based interventions, such as varnishes and toothpaste, strengthen enamel and reduce cavities. Dental sealants on molars provide added protection against decay. Oral hygiene education in schools helps children establish good brushing and flossing habits. Additionally, dietary modifications, such as reducing sugar intake and increasing calcium-rich foods support stronger teeth. Regular dental check-ups allow for early detection and management of potential issues (30).

India has implemented some initiatives to promote child health, such as the Rashtriya Bal Swasthya Karyakram (RBSK), which addresses various paediatric health concerns. However, dedicated national programs focusing specifically on paediatric dental care remain scarce. Strengthening school-based oral health programs and integrating dental services into primary healthcare could significantly improve outcomes. Fostering preventive care and early interventions can reduce the long-term burden of dental diseases and promote lifelong oral health (31).

### India's oral health: inadequate budget allocation

**Figure 3:**
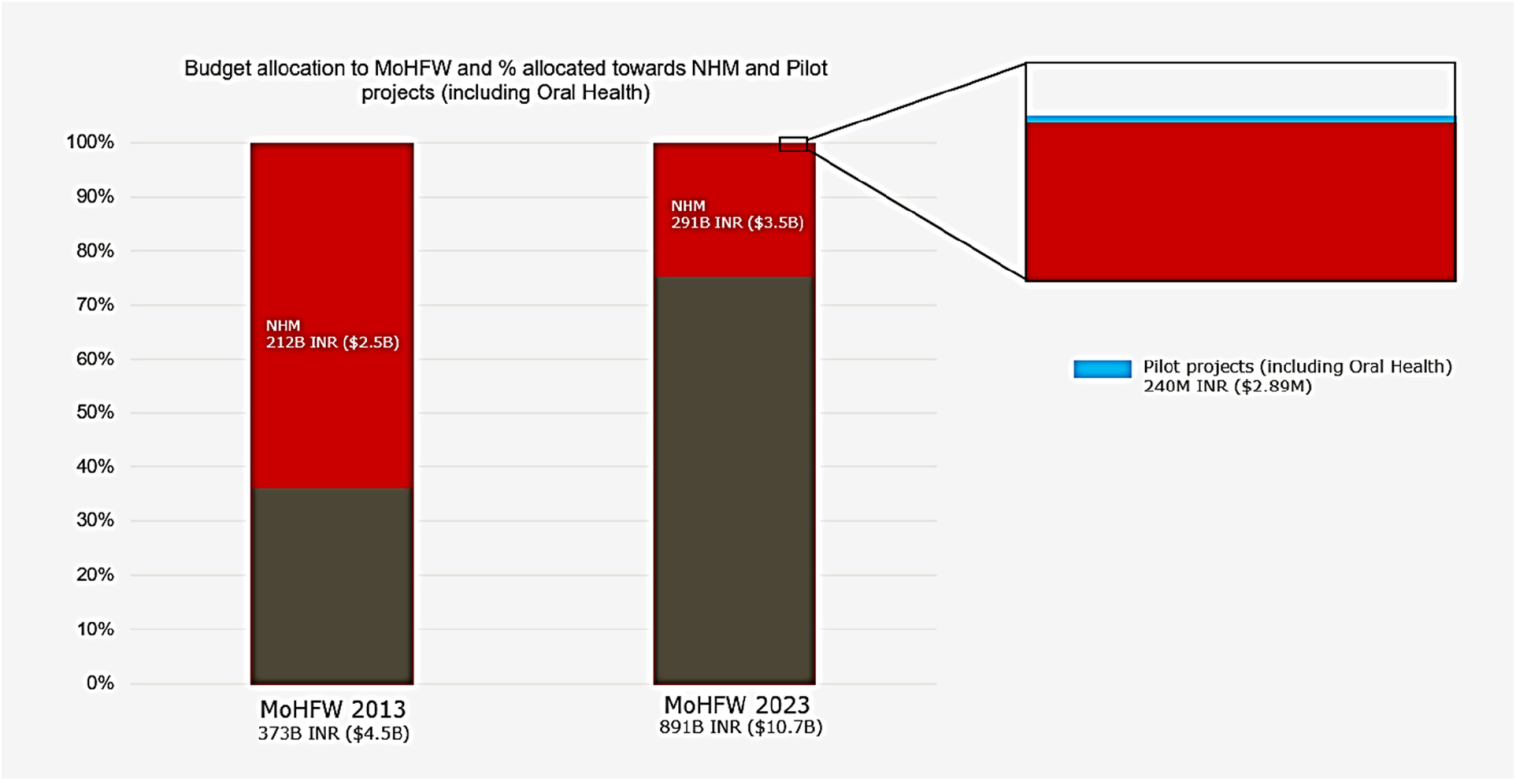
Budgets figures for the year 2013 and 2023. B: Billion; INR: Indian rupee; M: Million; MoHFW: Ministry of health and family welfare; NHM: National Health Mission.

India's public healthcare budgets is among the lowest globally (27). In 2013–14, the Indian government allocated 2.2% of its 16.65 trillion INR budget to the Ministry of Health & Family Welfare (MoHFW), with no specific funds earmarked for oral health (32). By 2023, the budget had increased to 45 trillion INR, with MoHFW receiving 2% (891.55 billion INR). Of this, only 240 million INR was designated to oral health under pilot schemes, equating to a per capita expenditure of just 5.4 INR. This allocation is negligible compared to other healthcare policies (Figure 3) (33). Additionally, out-of-pocket payments account for 62.6% of healthcare costs in India, compared to 10%–12% in the US, underscoring the urgent need for dedicated funding for oral health initiatives (34).

### Building a sustainable oral healthcare framework

Advocacy for improved access to care, oral health education, and basic infrastructure is urgently needed. Public-private partnerships could play a vital role in extending the reach of these initiatives and ensuring the establishment of sustainable oral health delivery systems. Incorporating oral health insurance into national health plans could significantly reduce out-of-pocket expenses and minimize productivity losses caused by oral health issues (35). Although national-level schemes like the Pradhan Mantri Jan Arogya Yojana and the Rashtriya Swasthya Bima Yojana provide health coverage, they currently do not include oral health services. Developing a public-private partnership model could enhance access and coverage by leveraging existing healthcare infrastructure (28).

Raising public awareness, empowering communities, and integrating oral health education into primary care are essential steps for building a sustainable oral healthcare framework (36). However, these efforts must be complemented by improving access to oral health services, as limited access often leads to treatable conditions worsening over time, resulting in long-term morbidity. Without timely and affordable care, minor dental issues such as cavities or gum disease can progress into severe complications, including tooth loss, chronic pain, and systemic health problems. This not only diminishes the quality of life for affected individuals but also places a heavier burden on healthcare systems (16). To address these challenges, public-private partnerships can play a transformative role by driving education, expanding the reach of health campaigns, and establishing sustainable delivery systems for oral health services (36). A glaring gap exists in up-to-date data collection on oral health indicators in India, as the last comprehensive survey was conducted in 2002. New surveys are urgently needed to provide current data and track progress effectively.

### Strengths and limitations

This study's strength lies in its integration of primary data from the OHO project combined with a systematic literature review, provides a well-rounded analysis of oral health behaviors, economic impact, and policy challenges in India. It is one of the few studies to evaluate the economic impact of oral diseases at both individual and national levels, highlighting the potential cost savings from preventive care. The study also highlights gender and age disparities in oral health, demonstrating variations in access, awareness, and financial burden. Additionally, the findings contribute to policy discussions by emphasizing the need for stronger interventions and public-private partnerships to improve oral healthcare infrastructure.

However, the study has certain limitations. A major limitation is selection bias, as the primary data comes exclusively from dental clinic attendees, potentially overlooking those who do not seek dental care, especially in rural areas. Additionally, the findings may not fully represent the national population due to regional disparities in oral healthcare access. The cross-sectional nature of the data prevents establishing causal relationships between oral health behaviors and economic burden. Furthermore, the study relies on existing literature but lacks recent, large-scale national data. While it identifies key policy challenges, its broad recommendations lack detailed implementation strategies, limiting practical impact.

### Future research and strategies

Future strategies to improve oral health in India should integrate policy reforms, public awareness campaigns, workforce expansion, and financial accessibility. Strengthening preventive care programs is essential to reducing reliance on emergency treatments through community-based screenings, school initiatives, and fluoride application programs (37, 38). Expanding public-private partnerships can enhance service delivery, particularly in underserved rural areas where access to professional dental care remains inadequate. Additionally, policy initiatives should prioritize integration of oral health services in national insurance schemes, encouraging routine visits and fostering regular preventive care (28, 39). Investing in mobile dental units and tele-dentistry can bridge accessibility gaps, especially in remote regions (40). Education and behavior change programs should be strengthened through digital platforms, mass media, and workplace wellness programs to promote regular oral hygiene habits (41).

To ensure long-term sustainability, research should focus on data-driven policymaking, including regular national oral health surveys to track progress and identify trends. Training and incentivizing mid-level dental providers can alleviate the burden on specialists while improving outreach. By implementing these strategies, India can transition from a reactive, treatment-focused model to a proactive, prevention-oriented oral healthcare system, reducing costs and improving overall health outcomes (42, 43).

## Conclusion

This study highlights the urgent need for preventive oral health strategies in India, emphasizing the shift from reactive treatments to proactive measures. Findings from the OHO project and secondary literature underscore critical gaps in oral health awareness, access to care, and economic burden. The decline in routine dental visits with age, particularly after 35–44 years, indicates a substantial need for early intervention programs to establish lifelong oral hygiene practices. The transition from partial to full edentulism in individuals aged 55–64 years further underscores the consequences of delayed preventive care. The economic burden of oral diseases, amounting to INR 613.2 billion (USD 7.3 billion) annually, is disproportionately high compared to the relatively low investment required for preventive care. Public-private partnerships and policy interventions can reduce treatment costs by promoting preventive measures, improving insurance coverage, and improving access to affordable dental services. Integrating oral health into national healthcare policies, such as the Pradhan Mantri Jan Arogya Yojana, can enhance accessibility and provide financial relief to underserved populations. Notably, gender disparities observed, with men reporting higher dissatisfaction and work-related disruptions due to oral pain, highlight the broader socioeconomic impact of inadequate oral healthcare.

In summary, future research should focus on population-based longitudinal studies to evaluate the cost-effectiveness of preventive interventions. Additionally, updated national oral health surveys are needed to inform policy decisions and track progress. Investing in preventive oral healthcare is not just a public health necessity but also an economic imperative for reducing long-term healthcare costs and improving the overall quality of life in India.
